# Effect of exercise alone and in combination with time-restricted eating on cognitive health in menopausal women

**DOI:** 10.3389/fpubh.2025.1640512

**Published:** 2025-08-13

**Authors:** Beata Jóźwiak, Paweł Kleka, Ida Laudańska-Krzemińska

**Affiliations:** ^1^Department of Physical Activity and Health Promotion Science, Poznan University of Physical Education, Poznań, Poland; ^2^Faculty of Psychology and Cognitive Status, Adam Mickiewicz University in Poznan, Poznań, Poland

**Keywords:** intermittent fasting, physical activity, menopause, cognition, BDNF, EEG

## Abstract

**Background:**

Cognitive complaints are commonly reported during menopause. Lifestyle interventions, such as exercise and time-restricted eating, are being investigated for their potential role in supporting cognitive health during menopause. This study investigated the effect of combining time-restricted eating (16:8) with a resistance and endurance circuit training program on cognitive health, as assessed using a comprehensive multi-domain cognitive test battery, in perimenopausal, menopausal, and postmenopausal women.

**Methods:**

Fifty-nine perimenopausal, menopausal, and postmenopausal women were assigned into a combination group (exercise + time-restricted eating, *n* = 26) and into an exercise group (exercise only, *n* = 33). Assessments were performed before and after a 12-week period and included Stroop Test, N-back Test, assessment of BDNF and GDNF level, and EEG testing.

**Results:**

Reading interference tendency in Stroop Test improved significantly in the exercise group (*p* = 0.026) while there were no changes in the combination group. Working time in Stroop Test decreased significantly in both groups (*p* = 0.025 – exercise group; *p* = 0.032 – combination group). Resting-state theta activity (eyes closed) and N-back test-related brain activity increased significantly only in the exercise group (*p* = 0.043). There were no significant changes in naming interference tendency in Stroop Test, N-back test results, BDNF level and GDNF level in any group.

**Conclusion:**

Combination of time-restricted eating and exercise does not result in superior improvements in cognitive health markers when compared with exercise alone in menopausal women. These results highlight the central role of physical exercise in maintaining cognitive health during menopause and suggest that adding time-restricted eating may not provide added value in this context. Future studies should investigate the underlying mechanisms through which multimodal lifestyle interventions may influence cognitive health in menopausal populations.

**Clinical trial registration:**

clinicaltrials.gov/study/NCT06138015, identifier NCT06138015.

## 1 Introduction

Menopause is a physiological transition that all women experience as a part of the aging process (1). During menopause, which typically occurs between the ages of 45 and 55, various metabolic (e.g., decreased glucose tolerance, insulin resistance, increased LDL, and decreased HDL) and somatic symptoms (e.g., hot flashes, heart palpitations) (2), as well as cognitive declines (e.g., poor working memory and attention span) (3–5) are observed. These cognitive changes are largely attributed to declining estrogen levels and their effects on neurotransmitter regulation (6). Accumulating evidence shows a significant neurotrophic and neuroprotective effect of estrogen on the central nervous system (CNS) (7). Estrogen modulates synaptic plasticity, regulates mitochondrial function and cerebral glucose metabolism, and reduces neuroinflammation – all of which are crucial for cognitive health (8). Cognitive deficits during the menopausal transition are particularly evident in domains such as working memory, attention, processing speed, and verbal memory (9, 10). In the Study of Women’s Health Across the Nation (SWAN), subjective memory complaints were reported by 31% of premenopausal women, compared to 44% in early perimenopause, 41% in late perimenopause, and 41% in postmenopause (11). Early spontaneous menopause onset has been linked to an increased risk of dementia and Alzheimer’s disease (AD) (12). These findings emphasize the need for effective, non-pharmacological interventions to support cognitive health during and after the menopausal transition. Lifestyle interventions – such as regular exercise and dietary changes – can help alleviate menopausal symptoms (2, 13), and support cognitive function (3) during this transition.

One promising lifestyle intervention that has gained attention and popularity is time-restricted eating (TRE). Time-restricted eating is a form of intermittent fasting (IF) that involves consuming meals within a limited time window, followed by a fasting period. Depending on the specific approach, the eating window can range from 6 to 12 h, with an average of 8 h (14). In recent years, IF has attracted growing interest from researchers due to its health and metabolic benefits, which are comparable to those of regular physical activity (15–17). While IF and exercise have been associated with improvements in cognitive function in various populations (14), evidence specific to middle-aged or menopausal women remains limited. The specific mechanisms underlying these cognitive benefits also remain unclear and are the focus of ongoing investigation.

The mechanisms by which IF affects brain function, remain under investigation. Animal models have provided initial evidence that IF may improve cognitive function and protect against neurodegenerative processes. Animal studies have demonstrated that IF exerts effects in models of brain-related diseases (18–20), though clinical research in this area remains limited. Current evidence suggests that IF does not yield short-term cognitive benefits in healthy individuals (21). However, emerging findings indicate that IF may offer protective effects against the development of neurological disorders (14). TRE shows promise as a preventive strategy for AD. A 4-month TRE intervention, involving an 8 h eating window from 8:00 AM to 4:00 PM, was found to enhance cognitive performance in patients with AD (22). Additionally, a form of intermittent fasting practiced for 3 years—from sunrise to sunset on Mondays and Thursdays—was associated with improved cognitive function and overall health in older adults with mild cognitive impairment (MCI), compared to individuals who practiced IF irregularly or not at all (23). Despite these findings, there is a lack of clinical studies investigating how IF impacts cognition in menopausal women – a population at elevated risk for neurodegeneration.

There is a compelling need to investigate the effects of IF in menopausal women, particularly in relation to cognitive decline and CNS disorders such as AD. IF may benefit cognitive health by stimulating autophagy, improving metabolic health, and restoring circadian rhythm regulation – both of which are altered in menopause. IF is one of the most effective inducers of autophagy – a cellular process responsible for degrading and recycling damaged components—which is essential for maintaining neuronal homeostasis and preventing neurodegeneration (24). In addition, IF has been shown to restore the expression of genes involved in the regulation of circadian rhythms (25), disruptions of which are recognized as significant risk factors for neurodegenerative diseases (26). IF is also known to improve cardiometabolic health (27). Women undergoing menopause face increased vulnerability to developing AD in later life, not only due to the loss of the neuroprotective effects of sex hormones, but also to lifelong hormonal influences and inherent sex-specific differences in the expression of autophagy-related proteins and circadian regulators (26). Moreover, menopause is associated with an increased risk of developing metabolic syndrome, which encompassess insulin resistance, visceral obesity, and dyslipidemia – conditions that may further contribute to a higher risk of developing AD in later life (28). Since AD is typically diagnosed after the age of 65, menopause may represent a modulatory factor influencing long-term risk rather than serving as a direct cause (29). Sex hormones modulate key critical biological processes, including circadian rhythm and autophagy, which may lead to sex-specific responses to dietary interventions such as IF (30). Therefore, targeted research is essential to evaluate the efficacy and safety of IF in menopausal women, considering their unique hormonal and neurobiological profiles.

In addition to dietary interventions such as IF, physical activity has also been recognized as a key non-pharmacological factor in the prevention of cognitive decline. A systematic review by Simmons et al. (31) reported that most studies examining this relationship found a significant negative association between leisure-time physical activity or physical fitness during the perimenopausal period and the risk of developing dementia later in life. Additionally, higher levels of household activity and non-leisure physical activity during this period were also linked to reduced dementia risk. These findings support the idea that physical activity may offer protective benefits against dementia after menopause.

The beneficial effects of PA and IF may be partly mediated by brain-derived neurotrophic factor (BDNF) and glial cell line-derived neurotrophic factor (GDNF). BDNF and GDNF are neurotrophins present in the adult brain that are essential for regeneration, maintenance, and survival of specific neuronal populations. Both play key roles in the pathogenesis of nervous system disorders, and alterations in their levels have been linked to cognitive decline (32). Among the various molecules that modulate cortical activity and influence short-term and working memory, BDNF is one of the most extensively studied, particularly in relation to physical activity (33). BDNF is critical for synaptogenesis (the formation of synapses), synaptic plasticity (the ability of synapses to strengthen or weaken in response to activity patterns), and neurogenesis (the process of generating new neurons) (34). It also supports cognitive abilities, such as memory and learning (35) and exerts neuroprotective effect, shielding neurons from various forms of damage – an important factor in the context of neurodegenerative and neuropsychiatric disorders (36). Given that estrogen regulates the expression of neurotrophic factors, including BDNF, further research is needed to explore the role of BDNF in menopause (37). GDNF plays a critical role in supporting neuronal survival, particularly that of dopaminergic neurons, which is especially relevant in the context of neurodegenerative diseases such as Parkinson’s disease (38). GDNF also promotes axonal regeneration and enhances neuroplasticity – the brain’s ability to adapt structurally and functionally—while protecting neurons from damage caused by toxic insults and oxidative stress (39). Owing to its neuroprotective and regenerative properties, GDNF is currently being investigated as a potential therapy for a range of neurological and psychiatric disorders (38, 40, 41). Preclinical studies have shown that ovariectomy—a commonly used model of menopause in mice—leads to a reduction in the expression levels of both BDNF and GDNF (32). These findings highlight the need to investigate strategies that modulate the levels of these neurotrophins, as their distribution may be disrupted in menopausal women.

Previous research has demonstrated that a 12-week combined resistance and aerobic training program significantly increased BDNF concentrations in both premenopausal and postmenopausal women with obesity, with a more pronounced effect observed in postmenopausal participants (42). However, evidence on changes in resting BDNF levels remains inconsistent across studies. A meta-analysis by Dinoff et al. (43) found that aerobic training, but not resistance training, was associated with increased resting concentrations of peripheral BDNF, while factors such as age, sex, and BMI did not significantly moderate this effect. The findings suggest that the type of exercise intervention plays a key role in BDNF regulation and that the observed effects may vary across studies and populations. Therefore, while there is evidence supporting the potential of physical training to modulate BDNF, further research is needed to clarify under what conditions resting BDNF levels reliably increase. BDNF has also been identified as a key mediator of enhanced glucose metabolism in response to exercise (44), suggesting potential benefits for mitigating metabolic risk factors commonly associated with menopause (45). Emerging evidence indicates that IF exerts pro-cognitive effects in conditions characterized by impaired BDNF signaling (46). Additionally, IF has been shown to protect against neuronal damage in animal models with estrogen deficiency, such as ovariectomized rats, where IF led to an increase in BDNF levels (47). Research on the effects of physical activity on GDNF levels in humans remains limited. One study reported that lower-body resistance training led to increased GDNF expression in skeletal muscle (48). Another study found that an 8-week Nordic Walking intervention had no effect on GDNF levels in postmenopausal women (49). This limited and inconsistent evidence highlights the need for further research to better understand the role of physical activity in modulating GDNF in humans.

Previous studies have shown that combining exercise with IF is more effective for weight loss and preserving muscle mass compared to exercise alone (50). In women with obesity, an 8-week intervention combining high-intensity interval training (HIIT) – performed as a circuit three times per week for 25 min per session – with an intermittent fasting (IF) protocol (a 5:2 diet, including two meals within a 6 h window, providing 25% of total energy intake, followed by 18 h of complete fasting) has been shown to be more effective for weight loss and muscle mass preservation than exercise alone (51). Similar results were observed in active women who followed a protocol combining isocaloric intermittent fasting with a 10 h eating window and 14 h of fasting, during which breakfast was consumed as soon as possible after waking. The fasting regimen was applied every other day and was paired with HIIT performed twice weekly, with each session lasting 40 min (52). However, findings from animal model suggest that this combination may not produce additional benefits for BDNF levels beyond those achieved by exercise alone. Specifically, in ovariectomized rats exercise alone was as effective as the combined intervention in increasing BDNF levels (47).

A promising method for assessing cognitive function involves the use of electroencephalography (EEG), which provides valuable insights into the brain’s neurophysiological activity (53). When combined with neuropsychological assessments, such as cognitive testing, EEG can offer a more comprehensive understanding of potential cognitive improvements following exercise or dietary interventions. Menopause has been shown to affect the brain’s bioelectrical activity. In early postmenopausal women, Beta2 power has been positively correlated with follicle-stimulating hormone (FSH) levels compared to premenopausal women, suggesting that FSH may influence fast cortical activation – a state associated with heightened cortical arousal and cognitive engagement—during the early postmenopausal period (54). Elevated beta EEG activity in late perimenopausal and postmenopausal women suggests that arousal level during sleep are higher in these groups (55). Moreover, beta EEG power provides an objective measure of hot flashes during the night, which have been shown to be frequently associated with sleep awakenings (56).

Although the positive effects of physical activity and IF on general health in humans are well established, no clinical studies to date have examined the combined impact of IF and exercise on cognitive function in menopausal women. Thus, it remains unknown whether TRE combined with exercise can modify or enhance cognitive health improvements in menopausal women compared to exercise alone. Taking this into account, the aim of the present study was to investigate whether the implementation of exercise and TRE, as opposed to exercise alone, has a significant impact on cognition in women undergoing menopause.

## 2 Methods

### 2.1 Primary and secondary outcomes

The primary outcome measures were the changes from baseline to week 12 in brain-derived neurotrophic factor (BDNF) levels, glial cell line-derived neurotrophic factor (GDNF) levels, N-back test performance, reading interference tendency (RIT) and naming interference tendency (NIT) in the Stroop test, and working time (WT) in the Stroop test.

The secondary outcome measures included changes from baseline to week 12 in brain wave activity, reaction time in the N-back test, accuracy in the N-back test, and Ruffier test.

### 2.2 Participants

**Figure 1:**
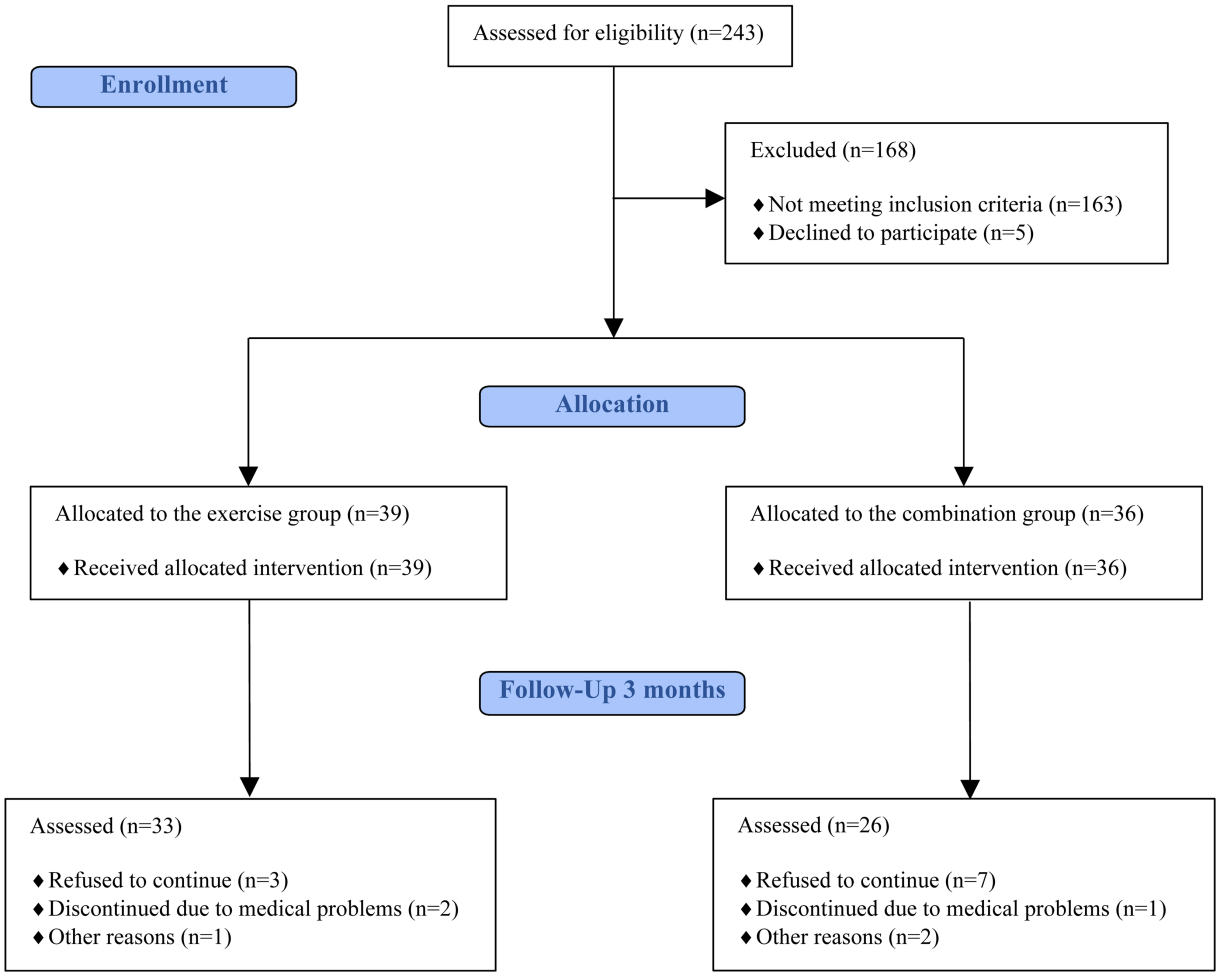
Flow diagram of the study following CONSORT guidelines.

Sample size calculations (G*Power 3.1.7) revealed that a minimum sample size of 54 participants would be appropriate to detect significant differences between the groups, assuming an effect size η_p_^2^ = 0.14, a type I error of 0.05, and a power of 0.80. On the basis of a prediction of a dropout rate of 20%, an estimated 68 were required to achieve a total of 54 participants. Participants were recruited in Poznań, Poland. Key inclusion criteria were: age between 41 and 61 years; perimenopausal status (defined as irregular menstrual bleeding within the last 12 months, including variability in cycle length of 7 days or more between consecutive cycles), menopausal status (amenorrhea for the past 12 months), or postmenopausal status (no menstrual bleeding for more than 1 year); and being lightly active or inactive (i.e., engaging in less than 3 h per week of light-intensity exercise at 2.5–4.0 metabolic equivalents [METs]). Physical activity levels were assessed using the Global Physical Activity Questionnaire (GPAQ) (57). Exclusion criteria included: use of hormone replacement therapy at any time or hormonal contraception; a history of hysterectomy, oophorectomy, or cancer treatment; presence of cardiovascular disease or type 1 or type 2 diabetes mellitus; use of antihypertensive, glucose-lowering, or lipid-lowering medications; body mass variation ≥ 4 kg in the past 3 months; a history of eating disorders; or working night shifts. Individuals with contraindications to physical exercise or fasting were excluded based on evaluation by an experienced physician. Of the 243 individuals who expressed interest in participating, 80 met the eligibility criteria (Figure 1). The experimental protocol was approved by the Bioethical Committee of the Poznan University of Medical Sciences (approval no. KB-179/21) and was conducted in accordance with the Declaration of Helsinki (58). Written informed consent was obtained from all participants prior to study participation. The experiment was registered at ClinicalTrials.gov (identifier: NCT06138015).

### 2.3 Study design and procedures

A 12-week quasi-experimental trial was conducted to evaluate the effects of exercise alone (exercise group), and TRE combined with exercise (combination group) on cognitive function in menopausal women. Eligible participants were assigned to one of two groups: (1) the combination group, which received both dietary and exercise interventions, and (2) the exercise group, which participated in the exercise intervention only. The trial was conducted in three consecutive waves: the first from October to December 2021, the second from January to March 2022, and the third from October to December 2022. Participant recruitment occurred over a 4-week period preceding each trial wave and was carried out through advertisements posted on the university website. During the second and third waves, additional participants were allocated to groups to ensure a balanced distribution and comparable group sizes by the end of the study.

#### 2.3.1 Collection and analysis of blood samples

Blood samples were collected by an experienced nurse both before the intervention and after its completion. A volume of 5 mL of blood was collected. The blood was left to clot at room temperature for 60 min (59) and centrifuged to obtain serum, which was subsequently deep-frozen at −80°C. Biochemical analyses were performed at the Molecular Endocrinology Laboratory of the Poznan University of Medical Sciences by experienced laboratory technicians. A total of 40 samples were collected, of which 37 were successfully analyzed.

ELISA kits were utilized to analyse Human BDNF (Sunredbio, Shanghai, catalog no. 201-12-1303) and Human GDNF (Sunredbio, Shanghai, catalog no. 201-12-0123), following the manufacturer’s instructions. All measurements were performed in duplicate, and the results were reported as the average of the two values.

#### 2.3.2 Cognitive ability assessment

The Stroop test was used to examine participants’ processing speed and selective attention (60). The test was a part of Vienna Test System. Two conditions were used without interfering influences (congruent stimuli) to determine baseline performance and were related to the two interference conditions, i.e., “color naming interference” and “word reading interference” (incongruent stimuli). Participants had to press the button of the appropriate color in the test panel as quickly as possible. The test took approximately 10 min and was preceded by familiarization trials for each condition. The system provided feedback to the participants in case of incorrect answers and did not allow them to proceed to the testing session until the correct answer was given. The reading interference tendency, and naming interference tendency in seconds, the working time for all test sections in minutes were analyzed. Reading interference tendency is a difference in reaction time between reading “incongruent” and reading “congruent.” Naming interference tendency is a difference in reaction time between naming “incongruent” and naming “congruent.” Lower values of reading interference tendency and naming interference tendency indicate better performance. Shorter working time across test section indicates better performance.

The N-back test was used to examine participants’ working memory, verbal memory, and attention (61). The test was a part of Emotiv Lab system. A sequence of graphics was presented to the participants at a fixed time interval. They were required to press a button whenever they noticed the same graphic that was shown two graphics earlier. The test took approximately 5 min and was preceded by written instruction. Data regarding accuracy and reaction time were collected and calculated to provide results in points. Better performance is reflected by higher total points, higher accuracy, and lower reaction time.

Emotiv EPOC X device was used to perform EEG testing. This device has been successfully employed in numerous research studies (62). It features 14 saline-soaked felt electrodes and two reference sensors, placed according to the 10–10 international system of EEG electrode placement at AF3, F7, F3, FC5, T7, P7, O1, O2, P8, T8, FC6, F4, F8, and AF4, and reference sensors at P3, P4. The interval sampling rate of the device is 2048 Hz, down sampled to 128 Hz. Measurement resolution was 14 bits with 1LSB = 0,51 μV (16-bit-1 DC, 2 bits instrumental noise floor discarded). Bandwidth range was 0.16–43 Hz with digital notch filters at 50 Hz and 60 Hz, and a dynamic range of 8,400 μV (pp). Participants were instructed to sit comfortably in a chair, after which the EEG headband was placed on their head and adjusted to ensure high contact quality. They were asked to minimize head and facial movements to reduce artifacts. The resting-state EEG recording lasted 4 min. During the first 2 min, participants kept their eyes opened while fixating on a cross displayed at the center of the computer screen. In the following, they sat with their eyes closed. Subsequently, EEG recordings were conducted during the performance of the N-back task, which was employed to induce external cognitive load, including working memory demands. In this task, participants were required to monitor a sequence of symbols presented on a computer screen and were instructed to press the space bar when the current symbol matched the one presented two positions earlier (2-back condition), immediately following the presentation of the current stimulus. The EEG device recorded brain activity across multiple frequency bands (theta, alpha, low beta, high beta, gamma), along with data on blinks, lower and upper facial movements, and head position changes detected via accelerometer. Data were transmitted to the EmotivPRO application via Bluetooth. Data were cleaned and artifacts were removed before further analysis. Absolute mean power for each frequency band was calculated by averaging across all sensors. A subset of participants underwent EEG testing. Data were collected from 16 individuals; however, recordings from 9 participants met the quality threshold in further analysis.

#### 2.3.3 Cardiorespiratory fitness assessment

The Ruffier test was administered before and after the interventions. The pretest heart rate (HR1) was measured while participants were standing, following five-minute rest period in a seated position. Participants were then instructed to perform 30 squats within 60 s. Each repetition consisted of two movements: squatting down to a 90-degree knee flexion and returning to a standing position. After completing the squats, the first post-test heart rate (HR2) was measured. The second post-test heart rate (HR3) was measured 60 s after completing the squats. The results of the test were calculated using the formula (HR1 + HR2 + HR3−200)/10.

### 2.4 Diet protocol

Only participants in the combination group took part in the dietary intervention, which required consuming all meals within an 8 h window or less, followed by fasting for the remainder of the day. The start time of the eating window was self-determined, and both the quantity and type of food consumed were chosen by participants. They were instructed not to change the type of foods they typically consumed. Nutritional data were collected weekly through an online survey, in which participants reported the time of their first and last meals each day. The exercise group was instructed to maintain their usual dietary habits. Before and after the intervention, all participants completed Dietary Habits, Lifestyle, Food Frequency Consumption and Nutritional Beliefs Questionnaire (KomPAN questionnaire) (63). As a Food Frequency Questionnaire (FFQ), KomPAN evaluates the food frequency consumption (FFC), which provides information about frequency of consumption of 33 different items, habitual consumption of foods, specific food components or nutrients, and dietary patterns. Ten of 33 items were classified as components of Healthy Diet Index and used to calculate Pro-Healthy Diet Index and fourteen of these items were classified as Unhealthy Diet Index and used to calculate Non-Healthy Diet Index.

### 2.5 Exercise protocol

Participants from both the combination and exercise groups took part in a supervised exercise intervention. The program consisted of moderate-intensity strength and endurance circuit training conducted twice weekly over a 12-week period. Exercises were performed using eight machines from the MILON system in a fixed sequence: cycle ergometer, abdominal crunch, leg curl, latissimus pulldown, elliptical machine, cross trainer, back extension, and leg abductor. Each strength exercise lasted 1 min, endurance bouts lasted 4 min, and a 30 s rest was provided between each activity. Each training session included three complete circuits of the sequence. Training intensity was estimated for each individual using an age-predicted heart rate maximum (HRmax) equation using Fox‘s equation (Fox-HR_max_ = 220 − age) (64). During each training session, a Polar Heart Rate Monitor (Polar USA, Inc., NY) was used to monitor each participants’ heart rate (HR). The intensity load in watts was adapted based on the monitored HR. According to the American College of Sports Medicine (ACSM), moderate intensity aerobic exercise is reached once a person’s heart rate reaches 64–76% of their age predicted maximal heart rate (65). The intensity of the resistance training was individualized according to the muscle strength level of each participant, which was assessed by 1 repetition maximum (1RM). Training intensity was progressively increased at 4-week intervals. Resistance exercises were performed at 50% of one-repetition maximum (1RM) during weeks 1–4, 60% of 1RM during weeks 5–8, and 70% of 1RM during weeks 9–12. Each session lasted 55 min. Participants were instructed to maintain their usual daily physical activity outside of the training program. To be included in the final analysis, individuals were required to attend at least 90% of sessions (a minimum of 22 out of 24 total sessions); those who did not meet this criterion were excluded from the final analysis. The moderate-intensity combined aerobic and resistance training protocol was chosen based on evidence (66) and ACSM recommendations (67) recommending this type of interventions to effectively support cognitive function.

### 2.6 Statistical analysis

All statistical analyses were performed using STATISTICA 13.3 (StatSoft, Inc.). After testing the assumptions of normality by the Shapiro–Wilk test, the *T*-test was applied in cases of parametric distribution for intra-group comparisons and the pair *T*-test for comparison between groups. The Wilcoxon test was applied in cases of non-parametric distribution for intra-group comparisons and the Mann–Whitney U test for comparison between groups. For the Wilcoxon test and the Mann–Whitney U test the effect size was calculated according to a formula r = |z|/sqrt(N) and interpreted as follows: 0.10–0.29 small effect, 0.30–0.49 moderate effect or r ≥ 0.50 large effect (68, 69). The results are expressed as mean ± standard deviation or median with upper and lower quartiles. The ANOVA two-way repeated measures mixed model for intra- and intergroup comparisons was performed. When a significant effect was found, *post hoc* multiple comparisons were performed. The effect size for ANOVA was assessed using the partial eta-square (η^2^) and values of 0.01, 0.06, and 0.14 were interpreted as small, medium, and large effects, respectively. In all tests, a *p*-value of less than 0.05 was set as statistically significant.

## 3 Results

### 3.1 Participants

A total of 80 participants were recruited for this study, with an overall dropout rate of 26% by the end of 12 weeks (Figure 1). The dropouts were primarily attributed to personal circumstances of the participants and concerns related to the COVID-19 pandemic. A total of 59 participants completed the study (Figure 1). Average age was 51.37 ± 4.71, average BMI was 27.23. ± 5.54, average systolic blood pressure (SBP) was 132.08 ± 16.52, and average diastolic blood pressure (DBP) was 84.10 ± 8.94. In terms of education level, 88% participants had higher education and 12% had secondary education. There was no significant difference in the distribution of premenopausal+menopausal and postmenopausal participants between the combination and exercise group (*p* = 0.358). In the exercise group, 14 participants were perimenopausal+menopausal and 19 were postmenopausal with regard to the cognitive test results. In the combination group, 8 participants were perimenopausal+menopausal and 18 were postmenopausal. For BDNF and GDNF analyses, the exercise group included 9 perimenopausal+menopausal and 12 postmenopausal participants, while the combination group included 6 perimenopausal+menopausal and 10 postmenopausal participants.

### 3.2 Adherence to exercise and/or dietary intervention

A total of 24 sessions of the training were conducted, with both groups completing over 96% of sessions. Participants were allowed to miss a maximum 2 out of 24 total sessions. Exercise compliance was assessed by recording attendance at each supervised exercise session. If an exercise session was missed, the participant was required to make up for the missed session. Almost 80% of participants were exercising at moderate or higher intensity for at least 50% of time during sessions. There was no difference in exercise intensity between groups (*p* = 0.994). Dietary compliance was lower than exercise compliance, with the combination group adhering to their prescribed 8 h long eating window in over 62% of days and adhering to 9 h eating window in almost 87% of days. Prior to the experiment, eating window of participants was on average 12 h and 26 min long. KomPAN FFQ results were calculated to compare baseline intensity of pro-healthy and non-healthy characteristics and intensity at the end of the study in both groups. There was no significant difference in food frequency consumption characterized by pro-healthy (*p* = 0.231) and non-healthy (*p* = 0.088) intensity before and after 12-weeks intervention in the combination group which may indicate that quality and amount of food while having TRE remained unchanged comparing to a diet that participants have had before the experiment.

### 3.3 Cognitive tests

There were no baseline differences in cognitive function indicators between the combination and exercise groups. Total working time in Stroop test decreased significantly in both groups (moderate effect sizes) and reading interference tendency (RIT) in Stroop test decreased significantly in the exercise group after 12 weeks (moderate effect size) (Table 1). There was no group x time interaction in cognitive tests’ outcomes (Table 1). At baseline, there were no statistically significant differences in cognitive test outcomes between perimenopausal+menopausal and postmenopausal participants, either in the overall sample or within the exercise group. In the combination group, baseline differences were also non-significant, except for the N-back score and N-back accuracy, where perimenopausal+menopausal participants showed significantly higher values than postmenopausal participants (both *p* = 0.045). There were no significant differences in pre-post change scores in cognitive tests between perimenopausal+menopausal and postmenopausal participants within the exercise group (Table 2). In the combination group, significant differences were observed between these subgroups in Stroop test RIT (*p* = 0.043, moderate effect size), as well as in N-back test score and accuracy (*p* = 0.008, large effect size and *p* = 0.003, large effect size, respectively) (Table 2). No significant differences were found between the subgroups in other variables within the combination group (Table 2).

**Table 1:** The effect of TRE and/or exercise on results of cognitive tests and Ruffier test.

Variable	Exercise group (*n* = 33)		*p*, r	Combination group (*n* = 26)		*p*, r	ANOVA GxT
	Baseline	12 weeks		Baseline	12 weeks		*p,* η^2^
STROOP RIT [sec.]	0.08 (0.03, 0.11)	0.04 (0.02, 0.07)	0.026*, 0.387	0.06 (0.03, 0.12)	0.07 (0.04, 0.10)	0.594, 0.105	0.546, 0.006
STROOP NIT [sec.]	0.06 (0.03, 0.12)	0.06 (0.03, 0.08)	0.224, 0.218	0.08 (0.05, 0.11)	0.07 (0.05, 0.10)	0.929, 0.017	0.375, 0.014
STROOP WT (min)	4.02 (3.58, 4.45)	3.72 (3.45, 4.22)	0.026*, 0.393	4.15 (3.53, 4.60)	3.94 (3.52, 4.55)	0.031*, 0.423	0.933, <0.001
N-back [points]	41.89 ± 13.52	45.52 ± 14.79	0.096, 0.368	40.81 ± 15.25	48.50 ± 15.65	0.194, 0.300	0.975, <0.001
N-back RT [ms]	502.44 ± 50.95	508.46 ± 49.74	0.211, 0.359	520.88 ± 47.54	504.70 ± 48.82	0.336, 0.285	0.775, 0.003
N-back – Accuracy [%]	69.50 (63.00, 76.00)	74.50 (61.50, 80.00)	0.263, 0.310	73.50 (61.00, 78.00)	77.50 (69.50, 82.00)	0.187, 0.352	0.935, <0.001
Ruffier test [points]	8.10 (6.00, 12.00)	7.60 (4.90, 9.20)	0.045*, 0.384	8.00 (6.40, 11.20)	6.20 (4.00, 9.05)	0.098, 0.344	0.898, <0.001

Values are means ± SD or medians (quartiles); r (effect size for Wilcoxon test) 0.10–0.29 indicating small effect, 0.30–0.49 indicating moderate effect or r ≥ 0.50 indicating large effect; partial eta-squared (η^2^) ≥ 0.06, indicating a moderate or η^2^ ≥ 0.14 large effect; * *p* < 0.05; GxT, group x time interaction; RIT, reading interference tendency; NIT, naming interference tendency; WT, working time; RT, response time.

**Table 2:** Pre-post change scores by menopausal status in exercise and combination groups.

Variable	Exercise group		*p*, r	Combination group		*p*, r
	Perimenopausal + Menopausal	Postmenopausal		Perimenopausal + Menopausal	Postmenopausal	
Δ STROOP RIT [sec.]	−0.02 (−0.05, 0.02)	−0.03 (−0.08, 0.00)	0.466, 0.127	−0.05 (−0.08, −0.00)	0.01 (−0.02, 0.05)	0.043*, 0.398
Δ STROOP NIT [sec.]	−0.01 (−0.08, 0.01)	−0.01 (−0.04, 0.02)	0.648, 0.079	−0.01 (−0.06, 0.06)	0.01 (−0.03, 0.02)	0.677, 0.082
Δ STROOP WT (min)	−0.19 (−0.37, 0.00)	−0.20 (−0.38, 0.02)	0.610, 0.089	−0.07 (−0.41, 0.04)	−0.13 (−0.23, 0.03)	0.933, 0.016
*Δ* N-back [points]	−2.00 (−5.00, 2.00)	10.00 (−0.50, 16.50)	0.305, 0.284	−11.00 (−17.00, 0.00)	14.00 (6.50, 33.00)	0.008*, 0.707
Δ N-back RT [ms]	−12.00 (−58.00, −5.00)	−40.50 (−54.50, 27.00)	0.714, 0.101	12.00 (−22.00, 50.00)	−27.50 (−64.50, −7.00)	0.138, 0.397
Δ N-back – Accuracy [%]	−3.00 (−3.00, 1.00)	10.50 (−3.50, 18.00)	0.212, 0.345	−6.50 (−12.00, 1.00)	8.50 (7.00, 27.50)	0.003*, 0.776
Δ BDNF (ng/ml)	0.10 (−0.10, 0.10)	−0.10 (−1.00, 0.05)	0.286, 0.233	−0.00 (−0.20, 0.50)	0.10 (0.00, 0.80)	0.480, 0.176
Δ GDNF (ng/ml)	0.10 (−0.50, 0.70)	−0.35 (−0.65, 0.05)	0.165, 0.302	0.65 (0.10, 0.70)	−0.65 (−2.20, 0.40)	0.073, 0.447

Values are Medians (quartiles); Δ – post-test minus pre-test values; r (effect size for Mann–Whitney test) 0.10–0.29 indicating small effect, 0.30–0.49 indicating moderate effect or r ≥ 0.50 indicating large effect; * *p* < 0.05; RIT, reading interference tendency; NIT, naming interference tendency; WT, working time; RT, response time; BDNF, brain-derived neurotrophic factor; GDNF, glial cell line-derived neurotrophic factor.

### 3.4 Blood indicators concentration

No significant differences in BDNF or GDNF concentrations were observed between the groups at baseline. There were no significant changes in BDNF or GDNF levels after 12 weeks (Table 3). There were no significant differences in pre-post change scores in BDNF or GDNF between perimenopausal+menopausal and postmenopausal participants within the exercise and combination groups (Table 2).

**Table 3:** The effect of TRE and/or exercise on level of neurotrophic factors.

Variable	Exercise group (*n* = 21)		*p,* r	Combination group (*n* = 16)		*p,* r	ANOVA GxT
	Baseline	12 weeks		Baseline	12 weeks		*p,* η^2^
BDNF (ng/ml)	1.90 (1.30, 3.30)	1.90 (1.30, 2.70)	0.268, 0.254	1.60 (1.20, 3.50)	1.85 (1.30, 3.00)	0.346, 0.252	0.071, 0.090
GDNF (ng/ml)	4.10 (3.00, 8.10)	3.90 (2.50, 7.50)	0.533, 0.143	3.80 (2.70, 7.65)	3.60 (2.65, 9.20)	0.910, 0.029	0.562, 0.010

Values are Medians (quartiles); r (effect size for Wilcoxon test) 0.10–0.29 indicating small effect, 0.30–0.49 indicating moderate effect or r ≥ 0.50 indicating large effect; partial eta-squared (η^2^) ≥ 0.06, indicating a moderate or η^2^ ≥ 0.14 large effect; GxT, group x time interaction; BDNF, brain-derived neurotrophic factor; GDNF, glial cell line-derived neurotrophic factor.

### 3.5 EEG

There were no differences in mean power of brain waves at baseline between the groups. Mean power of theta wave increased significantly during eyes closed resting-state in the exercise group (large effect size) (Table 4). Mean power of theta, alpha, beta low, beta high, and gamma waves increased significantly during N-back test in the exercise group (large effect sizes) (Table 4). No significant changes were observed in the combination group regarding EEG testing (Table 4).

**Table 4:** The effect of TRE and/or exercise on brain waves activity.

Variable	Exercise group (*n* = 5)		*p, r*	Combination group (*n* = 4)		*p,* r
	Baseline	12 weeks		Baseline	12 weeks	
Theta EO	6.11 (3.12, 9.59)	44.69 (41.47, 50.35)	0.080, 0.784	3.07 (1.76, 7.63)	17.50 (6.82, 58.97)	0.068, 0.913
Alpha EO	2.14 (2.05, 3.31)	11.84 (11.37, 15.17)	0.080, 0.784	2.08 (1.09, 3.25)	5.80 (2.28, 19.85)	0.144, 0.730
Beta low EO	1.43 (1.11, 1.52)	4.56 (4.17, 7.36)	0.225, 0.543	1.14 (0.72, 1.54)	2.56 (1.20, 7.78)	0.068, 0.913
Beta high EO	0.59 (0.49, 1.06)	1.70 (1.32, 3.54)	0.138, 0.663	0.84 (0.52, 0.91)	1.10 (0.59, 3.20)	0.068, 0.913
Gamma EO	0.23 (0.20, 0.64)	1.38 (0.56, 1.48)	0.080, 0.784	0.36 (0.25, 0.49)	0.43 (0.27, 1.08)	0.068, 0.913
Theta EC	9.52 (5.07, 9.61)	41.58 (14.53, 45.90)	0.043*, 0.905	2.29 (1.35, 9.76)	7.13 (2.60, 20.68)	0.068, 0.913
Alpha EC	3.13 (2.97, 4.03)	12.32 (8.41, 16.73)	0.225, 0.543	4.59 (1.25, 7.21)	4.13 (1.36, 6.81)	1.000, 0.000
Beta low EC	1.39 (1.19, 3.16)	5.48 (4.54, 8.07)	0.225, 0.543	1.34 (0.69, 2.56)	1.53 (0.76, 2.14)	1.000, 0.000
Beta high EC	0.56 (0.46, 0.90)	1.93 (1.58, 2.98)	0.345, 0.422	0.95 (0.52, 1.51)	0.65 (0.38, 0.89)	0.273, 0.548
Gamma EC	0.23 (0.20, 0.39)	0.52 (0.43, 0.87)	0.500, 0.302	0.45 (0.19, 1.41)	0.23 (0.18, 0.33)	0.273, 0.548
Theta N-back	3.38 (3.18, 6.03)	27.10 (18.39, 30.30)	0.043*, 0.905	3.72 (1.76, 30.09)	35.28 (13.63, 72.55)	0.465, 0.365
Alpha N-back	1.91 (1.30, 1.99)	6.57 (3.86, 14.56)	0.043*, 0.905	2.17 (0.93, 7.59)	7.66 (4.21, 16.89)	0.465, 0.365
Beta low N-back	0.91 (0.72, 1.49)	3.16 (1.77, 7.06)	0.043*, 0.905	1.24 (0.63, 3.34)	3.20 (1.51, 7.14)	0.715, 0.183
Beta high N-back	0.44 (0.33, 0.94)	1.97 (0.84, 2.76)	0.043*, 0.905	0.77 (0.47, 1.39)	1.31 (0.80, 2.32)	0.715, 0.183
Gamma N-back	0.22 (0.18, 0.45)	1.05 (0.61, 4.02)	0.043*, 0.905	0.47 (0.33, 0.72)	0.54 (0.37, 0.70)	1.000, 0.000

Values are Medians (quartiles); * *p* < 0.05; r (effect size for Wilcoxon test) 0.10–0.29 indicating small effect, 0.30–0.49 indicating moderate effect or r ≥ 0.50 indicating large effect; EO, eyes opened; EC, eyes closed.

### 3.6 Cardiorespiratory fitness

There was no baseline difference in Ruffier test results between the combination and exercise groups. Ruffier test results decreased significantly in the exercise group (*p* = 0.045, moderate effect size) while there was no significant change in the combination group (Table 1). There was no group x time interaction in Ruffier test outcomes (Table 1).

## 4 Discussion

To our knowledge, this study is the first to examine the combined effects of intermittent fasting and exercise on cognition in menopausal women. The aim of the study was to determine whether implementing time-restricted eating alongside exercise, compared to exercise alone, would lead to significant cognitive improvements in menopausal women. Initially, we hypothesized that the combination of TRE and exercise would produce greater cognitive benefits than exercise alone. However, the main finding indicated that cognitive outcomes were generally similar between the two groups, suggesting that the observed changes were primarily driven by the exercise intervention rather than the dietary component. Nonetheless, in the absence of a passive control group, potential practice effects cannot be ruled out.

Declines in estrogen level during the menopausal transition have been linked to reductions in attention and memory (4). Intermittent fasting (IF) has primarily been studied for its effects on weight-loss, though it also appears to have positive effects against neurological disorders – potentially due to its impact on weight loss (14). To date, no study has directly compared the cognitive effects of IF between individuals with obesity and without obesity. It remains unclear whether the cognitive benefits of IF stem from direct effects on the brain or from general health improvements, such as enhanced insulin sensitivity or weight reduction (14). In the current study, the intervention’s effect on cognition in the combination group was modest; however, notably, exercise alone produced a significant cognitive benefit.

Campbell et al. (70) showed that 6-months intervention of 150 min of aerobic exercise per week resulted in no significant changes in Stroop test in postmenopausal women who were breast cancer survivors. Contrary to these results, in this study Stroop test results improved in the exercise group. It might be the result of choosing different form of physical activity and including not only postmenopausal women, but also perimenopausal and menopausal women. According to systematic review of Oliveira et al. (71), effect of high intensity-interval training is significant on Stroop test results, but moderate aerobic training does not provide such changes. In this study, exercise alone produced a significant improvement in Stroop test performance, despite being of moderate intensity. The addition of TRE did not result in further enhancement of Stroop test outcomes in the combination group.

In this study, cardiorespiratory fitness, as measured by the Ruffier test, improved significantly only in the exercise group. This improvement may help explain the cognitive benefits observed in this group. As noted by España-Irla et al. (72), the relationship between cardiorespiratory fitness and cognitive function is evident in healthy middle-aged adults. This association is mediated by brain structure, suggesting a potential mechanistic pathway through which enhanced cardiorespiratory fitness may positively influence cognitive performance during midlife.

Cognitive problems, including declines in working memory, attention and verbal memory, are common during perimenopause (73). Working memory, verbal memory and attentional processes play crucial role in the N-back task (61). Konishi et al. (74) observed that worse N-back test performance is associated with lower BDNF level in postmenopausal women only. In premenopausal and perimenopausal women there was no such association. In this study, there was no change in N-back test after intervention and no change in BDNF or GDNF level in any group. Selecting postmenopausal women for this study might have potentially led to greater changes in BDNF levels and N-back test performance following exercise and diet intervention. However, subgroup analysis revealed that menopausal status may influence the cognitive response to the intervention. There were no significant differences in pre-post changes in cognitive performance, BDNF, or GDNF between perimenopausal+menopausal and postmenopausal participants within the exercise group. In contrast, significant between-group differences emerged in the combination group. Specifically, Stroop test RIT increased in postmenopausal participants and decreased in perimenopausal+menopausal participants, suggesting a decline in selective attention in the former group and improvement in the latter. As higher Stroop RIT values reflect worse cognitive performance, these findings indicate that postmenopausal participants benefited less from the combined TRE and exercise intervention in terms of selective attention. Similarly, significant differences between subgroups were observed in the N-back performance after the intervention, while perimenopausal+menopausal women exhibited a decline. However, these results must be interpreted with caution, as baseline N-back scores and accuracy were significantly higher in the perimenopausal+menopausal subgroup, potentially leading to ceiling effects and confounding the interpretation of post-intervention changes. These findings suggest that cognitive responses to combined TRE and exercise interventions may vary depending on the menopausal stage, with postmenopausal women showing less pronounced improvements-particularly in domains such as selective attention. This underscores the importance of considering menopausal status when evaluating intervention outcomes and highlights the need for further research into the biological mechanisms that may underline these differential responses.

Previous studies have suggested that the cognitive effects of physical activity may differ by sex and genotype. Watts et al. (75) reported cognitive improvements associated with physical activity in men without the BDNF Val66Met polymorphism, whereas women showed no significant cognitive or BDNF level changes regardless of genotype. In line with these findings, our study, conducted exclusively in women, found no increase in BDNF levels following physical activity. It should be noted that the Val66Met polymorphism does not necessarily correlate with peripheral BDNF concentrations, suggesting that other mechanisms may contribute to sex-specific responses to exercise.

According to Tang et al. (76), increased theta wave power following meditation is associated with a relaxed state and reduced stress. In the present study, theta power significantly increased in the exercise group during resting-state EEG with eyes closed, suggesting a relaxation effect induced by physical exercise training. In contrast, no significant changes were observed in the combination group, implying that the stress-reducing effect may have been diminished when TRE was combined with exercise. Additionally, brain wave activity during the N-back task – a working memory-intensive task – was significantly elevated in the exercise group post-intervention compared to baseline. Increases in theta, alpha, beta, and gamma activity during this task may reflect enhanced support for working memory processes and improved cognitive performance in menopausal women who completed a 12-week physical training program. Physical activity is known to enhance perceptual and environmental processing demands, which can influence brain activity (77). Working memory is a core component of human cognitive functioning (78). Zheng et al. (79) reported that working memory performance improved during acute aerobic exercise in young adults, as measured by a modified N-back task, highlighting the potential of physical activity to enhance working memory function. Hsieh et al. (80) found no effect of acute exercise on brain wave activity. These findings, when considered alongside the current study, suggest that sustained, long-term exercise interventions may be necessary to produce measurable changes in EEG activity. Furthermore, the results highlight the importance of cognitive load during testing for detecting such neural adaptations. Notably, this is the first study to examine the effects of IF on EEG and it remains unclear why effect of exercise on brain waves activity was not present in the combination group.

In the study by Espeland et al. (81), a multidomain intensive lifestyle intervention – including both dietary changes and increased physical activity did not yield cognitive benefits for adults with type 2 diabetes who had baseline cognitive complaints. Initiating and managing two simultaneous health behavior changes may have been cognitively demanding and potentially stress-inducing. This could help explain why greater improvements in cognitive measures were observed in the exercise-only group, where participants focused on a single lifestyle modification.

Findings in the literature regarding fasting and other dietary interventions and their effects on cognition are inconsistent. Senderovich et al. (82) reported that while some studies have found cognitive improvements following calorie restriction, other evidence suggests that dietary interventions alone may not be sufficient, and that exercise may yield more consistent cognitive benefits. For example, Kim et al. (83) observed a significant decline in recognition memory performance among healthy adults with elevated waist circumference who followed an intermittent calorie restriction protocol (600 kcal for two consecutive days per week) for 4 weeks. In contrast, no change was observed in a group that followed continuous calorie restriction. Furthermore, review by Benau et al. (84) indicated that fasting can be associated with cognitive deficits, and that any cognitive benefits tend to occur after short-term, rather than long-term fasting. Since intermittent fasting is typically practiced as a long-term dietary approach, this may help explain the lack of cognitive improvements observed in the combination group compared to the exercise-only group in the present study.

This study has several limitations. First, the inclusion criteria may have introduced selection bias, with the wide range of menopausal age potentially acting as a confounding factor. Additionally, the classification of menopausal status in this study was based solely on self-reported menstrual history, without the use of hormone concentrations or the STRAW+10 staging system, which is considered the gold standard for precise menopausal staging. Future studies should incorporate the STRAW+10 criteria to enable more accurate and standardized assessment of menopausal status. Another limitation is the relatively short duration of the intervention – 12 weeks – which may limit the generalizability of the findings to long-term outcomes. Participant dropout was higher in the combination group compared to the exercise group, which may have introduced bias. The study did not include data on energy intake, limiting the ability to evaluate the potential role of caloric restriction in the observed effects. The timing of the eating windows was not standardized across participants; individuals were free to choose the start time of their eating window, resulting in variability. The study was conducted during COVID-19 pandemic, which associated restrictions that may have affected participants’ lifestyle, stress level, ad adherence to interventions. No corrections for multiple comparisons were applied, as EEG, cognitive, and neurotrophins outcomes were analyzed independently. Furthermore, EEG recordings were obtained using a consumer-grade device designed for research and commercial purposes rather than clinical diagnostics, and thus has inherent limitations. The use of per-protocol rather than an intent-to-treat analysis may limit the generalizability of the findings. Lastly, the sample size was limited. Further research with larger cohorts is necessary to better determine the optimal dosage of fasting and exercise for cognitive enhancement in menopausal women.

We demonstrated that combination of time-restricted eating and exercise does not result in superior improvements in cognitive health markers when compared with exercise alone in menopausal women. Combination of time-restricted eating and exercise might not be as effective strategy for prevention of cognitive decline among menopausal women as exercise alone. Further studies with larger study sample are needed to investigate effect of combined time-restricted eating and exercise on cognition in women during menopausal transition.

## Data Availability

The raw data supporting the conclusions of this article will be made available by the authors, without undue reservation.
